# A Novel Approach to Investigate the Effect of Tree Reconstruction Artifacts in Single-Gene Analysis Clarifies Opsin Evolution in Nonbilaterian Metazoans

**DOI:** 10.1093/gbe/evaa015

**Published:** 2020-02-03

**Authors:** James F Fleming, Roberto Feuda, Nicholas W Roberts, Davide Pisani

**Affiliations:** e1 School of Earth Sciences, University of Bristol, United Kingdom; e2 Faculty of Environment and Information Studies, Keio University, Tsuruoka, Yamagata, Japan; e3 School of Biological Sciences, University of Bristol, United Kingdom

**Keywords:** opsin, phylogeny, systematic error, nonbilaterian metazoan

## Abstract

Our ability to correctly reconstruct a phylogenetic tree is strongly affected by both systematic errors and the amount of phylogenetic signal in the data. Current approaches to tackle tree reconstruction artifacts, such as the use of parameter-rich models, do not translate readily to single-gene alignments. This, coupled with the limited amount of phylogenetic information contained in single-gene alignments, makes gene trees particularly difficult to reconstruct. Opsin phylogeny illustrates this problem clearly. Opsins are G-protein coupled receptors utilized in photoreceptive processes across Metazoa and their protein sequences are roughly 300 amino acids long. A number of incongruent opsin phylogenies have been published and opsin evolution remains poorly understood. Here, we present a novel approach, the canary sequence approach, to investigate and potentially circumvent errors in single-gene phylogenies. First, we demonstrate our approach using two well-understood cases of long-branch attraction in single-gene data sets, and simulations. After that, we apply our approach to a large collection of well-characterized opsins to clarify the relationships of the three main opsin subfamilies.

## Introduction

Resolving gene phylogenies is difficult for two reasons. Firstly, single-gene alignments are relatively short and might be poor in phylogenetic signal. Secondly, it is more difficult to counter tree reconstruction artifacts in single-gene alignments, as most approaches used to address these problems have been developed for long superalignments where such artifacts are exacerbated (Lartillot and Philippe 2004; Jeffroy et al. 2006)—for example, the CAT-based models of Lartillot and Philippe (2004) and methods that remove substitutionally saturated sites (e.g., Brinkmann and Philippe 1999; Pisani 2004; Sperling et al. 2009). However, in the postgenomic era, we have access to an abundance of sequences from a multitude of species for many gene families. Sequence-rich alignments can thus be subsampled to exclude “problematic sequences” (i.e. sequences that lack sufficient phylogenetic signal to be roboustly resolved under the considered substitution model – as suggested by the conclusions of Felsenstein (1978), Anderson and Swofford (2004), and Xi et al. (2015)). Problematic sequences are likely to have a distorting effect on gene trees, and their identification and exclusion could constitute an alternative, viable strategy to improve the accuracy of single-gene phylogenies. However, assessing how to objectively identify such sequences is far from trivial.

Opsins are G-coupled protein receptors fundamental to light sensitive processes across Metazoa. Opsins are present in almost every animal phylum; including Cnidaria and Ctenophora (Feuda et al. 2012; Schnitzler et al. 2012; Feuda et al. 2014; Ramirez et al. 2016). Opsin-like sequences have also been found in Placozoa, known as placopsins (Feuda et al. 2012, 2014), though opsins and opsin-like sequences are currently unknown in sponges (Plachetzki et al. 2007; Feuda et al. 2012). General agreement exists that most opsins can be ascribed to one of three “canonical” (i.e., widely recognized—see Ramirez et al. 2016) subfamilies: the rhabdomeric opsins, the ciliary opsins, and the group 4 opsins (the peropsins/RGRs, Go-opsins, and the neuropsins). In addition to these groups, Ramirez et al. (2016) defined three more opsin subfamilies: the bathyopsins, the xenopsins and the chaopsins, though the monophyly of these new families requires further testing.

Despite their physiological diversity, most opsins function in a similar fashion. They bind to a chromophore, an aldehyde derivative of vitamin A, in its *cis-*photoisomerized state. Together, this combination is known as a visual pigment (Terakita 2005). When the chromophore absorbs a photon of light, it changes from its *cis* state to its *trans* state, which alters the conformation of the opsin-chromophore binding site and activates the G-protein the opsin is coupled to, thus starting a signaling cascade (Terakita 2005). Rhabdomeric visual pigments are bistable: The chromophore stays attached to the opsin when in *cis* or *trans* state (Tsukamoto 2014). The chromophore is able to reversibly switch between the *cis* and *trans* conformational states by absorbing light of different wavelengths (Tsukamoto and Terakita 2010). However, in ciliary visual pigments, the chromophore becomes detached from the opsin when it changes conformation from *cis* to *trans*. This is known as opsin bleaching. RGRs bind to all-*trans*-retinal and convert the chromophore back to all-*cis*-retinal; that can then be reattached to bleached ciliary opsins (Terakita 2005). Visual ciliary opsins are therefore dependent on the presence of RGRs to function, although it is still unclear whether some nonvisual ciliary opsins undergo bleaching (Terakita et al. 2012).

**Fig. 1.**
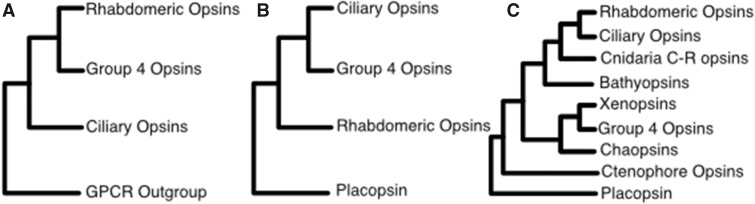
—Competing hypothesis on the phylogenetic affinities of canonical opsin families. (*A*) Porter et al. (2012); (*B*) Feuda et al. (2012, 2014) and Hering and Mayer (2014); (*C*) Ramirez et al. (2016).

The relationships among the canonical opsin families are still unclear (see fig. 1). Three main scenarios have been proposed for their evolution. The first scenario, proposed by Feuda et al. (2012, 2014), Hering and Mayer (2014), and Schnitzler et al. (2012), suggests a sister group relationship between the group 4 and the ciliary opsins to the exclusion of the rhabdomeric opsins. The second, proposed by Porter et al. (2012) found a sister group relationship between the group 4 and rhabdomeric opsins to the exclusion of the ciliary opsins (fig. 1). Finally, Ramirez et al. (2016) found a sister group relationship between the rhabdomeric and ciliary opsins to the exclusion of the group 4 opsins. All noncanonical opsin families nest within the group defined by the three canonical opsin families. Hence, the deepest duplication in the history of the animal visual opsins is defined by the split between the rhabdomeric opsins and either the ciliary or the group 4 opsins (fig. 1). Lack of consensus on the relationships between rhabdomeric, ciliary and group 4 opsins implies that the identity of the opsins that were part of the ancestral metazoan photoreceptive system remains unclear.

Here, we present a new approach—the canary sequence approach—which can be applied to single-gene phylogenies and aims to identify and remove potentially problematic sequences. The name of the method derives from the practice of using canaries to detect methane in mine shafts. The canary approach uses sequences that change position between multiple rounds of tree searches (our canaries), but do not affect the relationships inferred for the other sequences in the data set, to identify potentially problematic sequences.

We first demonstrate that our approach is able to identify potentially problematic sequences in two classic case studies—recovering a tree displaying Ecdysozoa using the data of Aguinaldo et al. (1997), and a tree assessing the monophyly of Platyhelminthes (and the relationships of the Lophotrochozoa) using the data of Carranza et al. (1997). Furthermore, we test the method using simulations. Finally, we focus on the cnidarian and ctenophoran opsins and on the three canonical opsin families (ciliary, rhabdomeric, and group 4), and use the canary sequence approach to investigate phylogenetic relationships at the root of the opsin tree . Our results corroborate those of Feuda et al. (2012, 2014) and Hering and Mayer (2014), and suggest that the deepest duplication in the history of the bilaterian opsins separates the rhabdomeric opsins from a group composed by the ciliary and the group 4 opsins. In addition, we confirm the existence of cnidarian rhabdomeric opsins, which emerge as the sister of the bilaterian rhabdomeric opsins. Although we could confirm the existence of cnidarian and ctenophoran opsins sharing a common ancestor with the ciliary opsins, we could not confirm the existence of cnidarian and ctenophoran opsins related to the group 4 opsins.

### The Canary Sequence Approach to Identify Problematic Sequences

**Fig. 2.**
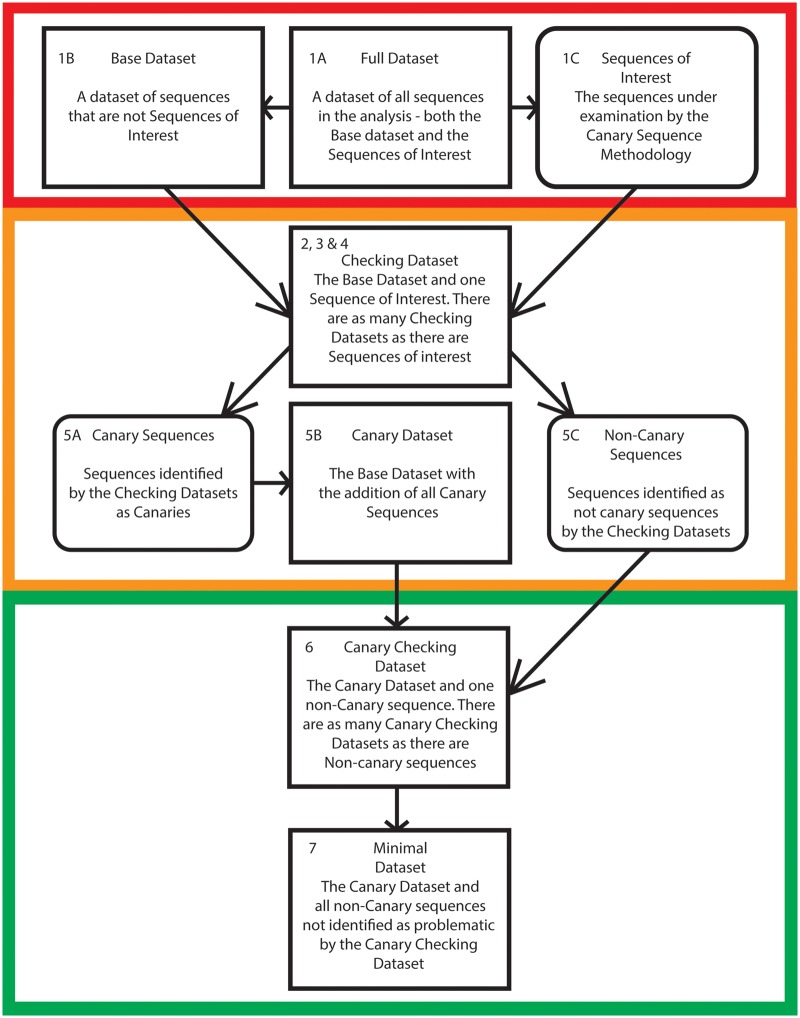
—A flowchart illustrating the key steps in the canary sequence approach. The first stage of the methodology (Red), shows how sequences are classified as members of the base data set or sequences of interest. In the second stage (Orange) sequences are assessed using checking data sets to determine whether or not they are canary sequences. In the fianl stage (Green) noncanary sequences are assessed using canary checking data sets to generate a minimal dataset and its associated minimal tree. Stages are numbered with reference to the steps in the pipeline described in the “The Canary Sequence Approach to Identify Problematic Sequences” section of this paper. A more detailed description of the methodology is available in supplementary figure S1, Supplementary Material online.

The canary sequence approach aims to identify and reduce the number of problematic sequences in an alignment, and thereby reduce topological reconstruction artefacts. The logic underlying the canary sequence approach is based on the identification of sequences that are prone to moving within a phylogeny due to poor clustering signals (Brinkmann and Philippe 1999; Dabert et al. 2010): The canary sequences. We then ascertain whether other sequences in the data set affect the phylogenetic relationships of the canary sequences to identify potentially problematic sequences. Potentially problematic sequences can then be excluded from the analyses in order to infer what we define as the “minimal tree” of a protein family (i.e. a gene tree that only includes the members of a gene family that are least likely to induce tree reconstruction errors). The steps of the canary method are presented in figure 2 and are summarized below:

1. *Data set creation*: The first step requires the identification of the “full data set” (the considered data set) and of two additional sub datasets. The first sub dataset is composed of the “sequences of interest,” which includes all the sequences that are under examination (these are a set of sequences that we intend to add to a pre-existing gene family data set). The second set is referred to as the “base data set,” which includes all sequences in the full data set except the sequences of interest. Trees are constructed from both the base data set and the full data set—these are referred to as the “base tree” and “full tree,” respectively. The base tree and full tree serve to measure the effect of the sequences of interest on the topology of the gene tree, and allow for an existing gene tree to act as a basis for the application of the canary method. Note that (see the results of our case studies) the method does not assume accuracy of either the base tree or full tree.
2. *Measuring the effect of the sequences of interest*: In the second step, a series of data sets are generated by separately combining the base data set with each individual sequence of interest. These data sets and the trees from these data sets are referred to as “checking data sets” and “checking trees.” The position of each sequence in each checking tree is noted.
3. *Identification of sequences for further examination*: For each sequence of interest, if the checking tree and the base tree are isomorphic (after the removal of the sequence of interest), the sequence of interest is marked as a “sequence for further examination.” If, after the removal of the sequence of interest, the checking tree and base tree are not isomorphic the sequence of interest is moved to the “noncanary sequences of interest data set.”
4. *Identification of canary and stable sequences:* As inaccurate phylogenies might emerge because of compositional heterogeneity (Roure et al. 2013), a posterior predictive test is performed to ascertains whether the “sequences for further examination” are compositionally homogeneous or heterogeneous. If a “sequence for further examination” is compositionally homogeneous and is found in different positions in the full tree and the checking tree, the sequence is identified as a “canary sequence.” Ifa sequence for further examination is compositionally homogeneous and found in the same position in the checking tree and the full tree , it is identified as a “stable sequence.” Any sequence for further examination that is found to be compositionally heterogeneous is moved to the “noncanary sequences of interest data set” irrespective of its placement in the chekcing, base and full tree.
  After each sequence of interest has been classified it is possible that canary sequences might not be present in a data set. If that is the case, move to step 8. If canary sequences are identified,, sequences previously identified as stable sequences are added to the “noncanary sequences of interest data set,” and the analysis moves to step 5.
  Steps 3 and 4 identify sequences that are unstable within their checking tree and have the expected amino acid composition. Such sequences do not have enough information to precisely cluster within their checking tree, but also do not convey enough clustering information to alter the relationships in the base tree (the compared trees are isomorphic once the canary sequence is removed). Because these sequences do not have sufficient information to cluster firmly in their checking tree, they are more likely to be affected by the presence of “problematic sequences” when compared with other sequences in the data set. We thus suggest that they can be used as indicators to highlight potentially problematic sequences, which are expected to have the tendency to attract canary sequences.
5. *Definition of the “canary data* *set”* and *“canary tree”:* All canary sequences identified in Step 4 are added to the base data set to generate the “canary data set.” A tree is inferred from the canary data set, which is referred to as the “canary tree.”
6. *Measuring the effect of the noncanary sequences on the canary data* *set*: For each sequence in the “noncanary sequences of interest data set”, a new alignment is generated where a single noncanary sequence of interest is added to the “canary data set.” These data sets and the trees they generate are referred to as the “canary checking data sets” and “canary checking trees” respectively. For each noncanary sequence of interest, if the “canary checking tree” and the “canary tree” (of step 5) are isomorphic (after the removal of the noncanary sequence of interest), the noncanary sequence of interest is identified as “nonproblematic.” All other noncanary sequences of interest are defined as “potentially problematic.”
7. *Generation of the “Minimal data* *set” and completion of the canary pipeline:* All “nonproblematic sequences” are added to the “canary data set” to generate the “minimal data set.” The tree generated from the minimal data set is the final point of the canary sequence approach and is called the “minimal tree.” This is to stress that this gene tree is by definition incomplete and only represents the backbone of the evolutionary history of the family of interest, as it excludes all potentially probelamtic sequences.
8. *No canary sequences Identified*: Previously identified “stable sequences” from step 3, are identified as potentially nonproblematic. Stable sequences only are added to the base data set to generate the minimal tree.

## Materials and Methods

To test the reliability of the canary approach we performed analyses using two data sets Aguinaldo et al. (1997) and Carranza et al. (1997) that address problems that were considered hard at the time these data sets were published, but that are now well understood. In addition to that, we used simulated data sets to further understand the behavior of the canary approach. Finally, we applied the canary approach to understand early opsin evolution. For both case studies (Aguinaldo et al. 1997; Carranza et al. 1997) and all simulation analyses, alignments were performed in MUSCLE (Edgar 2004) and analyzed under the JC69 model in PhyML (Guindon et al. 2010). JC69 was used to generate results comparable to those of the original studies, which did not have access to parameter rich models available today.

### Case Study 1

We used the Aguinaldo and collaborators 18s rRNA data set to test the performance of the canary method. The original 18s rRNA analysis of Aguinaldo et al. (1997) recovered a monophyletic Ecdysozoa through increased sampling of the Nematoda. We selected this data set as it represents a key study solving what is now accepted as a notable long-branch attraction artefact. Here, we tested whether the canary method was able to recover the monophyly of Ecdysozoa by removing problematic sequences. In this experiment the nematode sequences were designated as “sequences of interest,” as these sequences were the focus of the Aguinaldo et al. (1997) study. Following the canary sequence approach (see fig. 2), after the construction of the “base data set” and the “full data set” and their respective trees, three “checking data sets” were generated, each consisting of 46 18S rRNA sequences—the “base data set” sequences plus one nematode sequence of interest. Every compositionally homogenous nematode sequence (i.e., sequence of interest) that resolved in a different phylogenetic position in the “checking tree” and the “full tree” (with the checking and base trees isomorphic after removal of the sequence of interest), was selected as a canary sequence. 

Once canary sequences were identified, they were added to the “base data set” to form the “canary data set,” which contained 47 sequences, and the sequence that was not determined to be a canary sequence was moved to the noncanary sequences data set. As there was only one noncanary sequence of interest one “canary checking data set” was constructed, consisting of 48 sequences. The “canary checking tree” was compared with the “canary tree” (see point 5 of the “The Canary Sequence Approach to Identify Problematic Sequences” section) to evaluate whether the noncanary sequence of interest was “potentially problematic” or not, and whether it was to be excluded from the “minimal data set” and the “minimal tree” that we built to complete the canary pipeline (see point 6 of the “The Canary Sequence Approach to Identify Problematic Sequences” section).

### Case Study 2

The original 18s rRNA analysis of Carranza et al. (1997) was unable to recover a monophyletic Platyhelminthes (inclusive of Catenulida) despite increased sampling of the Platyhelminthes. Here, we used the Carranza et al. (1997) data set to attempt to establish whether a monophyletic Platyhelminthes could instead be recovered through application of the canary sequence approach. This data set was chosen because both the “full tree” and “base tree” (point 1 above) do not conform to modern understandings of platyhelminth relationships. Accordingly, this test allowed us to evaluate the extent to which the canary approach is robust to the use of an inaccurate “base tree” to identify canary and noncanary sequences.

We started by considering all 15 platyhelminth sequences in the data set as “sequences of interest,” as these sequences were the focus of Carranza et al. (1997) study. We defined the “base data set” as the complete data set of Carranza et al. (1997), the “full data set,” minus the platyhelminth sequences. We then generated 15 checking data sets, each consisting of 16 species—the base data set plus one sequence of interest (as in point 2 of the “The Canary Sequence Approach to Identify Problematic Sequences” section). We followed the rules in points 2–4 of the “The Canary Sequence Approach to Identify Problematic Sequences” section to partition the sequence of interest in “canary sequences” and “non-canary sequences of interest.”

Once canary sequences were identified, they were added to the base data set to generate the canary data set (point 3 of the "The Canary Sequence Approach to Identify Problematic Sequences" section), which contained 16 sequences. We identified 14 noncanary sequences of interest , and we thus generated 14 “canary checking data sets” consisting of 17 sequences each—the canary data set plus one noncanary sequence of interest. The 14 “canary checking trees” were compared with the “canary tree” to identify the “potentially problematic sequences”, generate the “minimal data set” and conclude the canary approach through the inference of a minimal tree (see point 6 of the "The Canary Sequence Approach to Identify Problematic Sequences" section).

### Simulation Data Sets

Fifty simulation data sets were constructed in PAML evolver (Yang 2007), using the Aguinaldo et al. (1997) data set and the Rev model. Each data set therefore included 49 sequences 1,968 nucleotides long—where 1,956 was the length of the shortest sequence in the Aguinaldo et al. (1997) data set. However, we increased the length of the long-branched sequences by 250% to further exacerbate long-branch attraction artifacts and increase the number of data sets where a standard phylogenetic analysis would be expected to recover an incorrect tree. This made the two long branches (*Strongyloides* and *Caenorhabditis*) ∼10 times longer than the next longest branches in the simulation. For each simulated data set we recovered trees using the JC69 model, to increase chances of recovering an incorrect topology, which we identified as any incorrect arrangement of nematode species (i.e., all cases where nematodes were not monophyletic or not members of Ecdysozoa). Simulated data sets that did not recover an incorrect topology, where no canary could be identified or where all sequences emerged as canary sequences, were not further considered as we only wanted to evaluate the number of successes in cases in which the full, standard, canary pipeline could be applied (points 1–6 of the “The Canary Sequence Approach to Identify Problematic Sequences” section). A success in the application of the canary approach was defined as the recovery of a monophyletic Nematoda as a memebr of the nonarthropod Ecdysozoa.

### Opsin Data Set

**Fig. 3.**
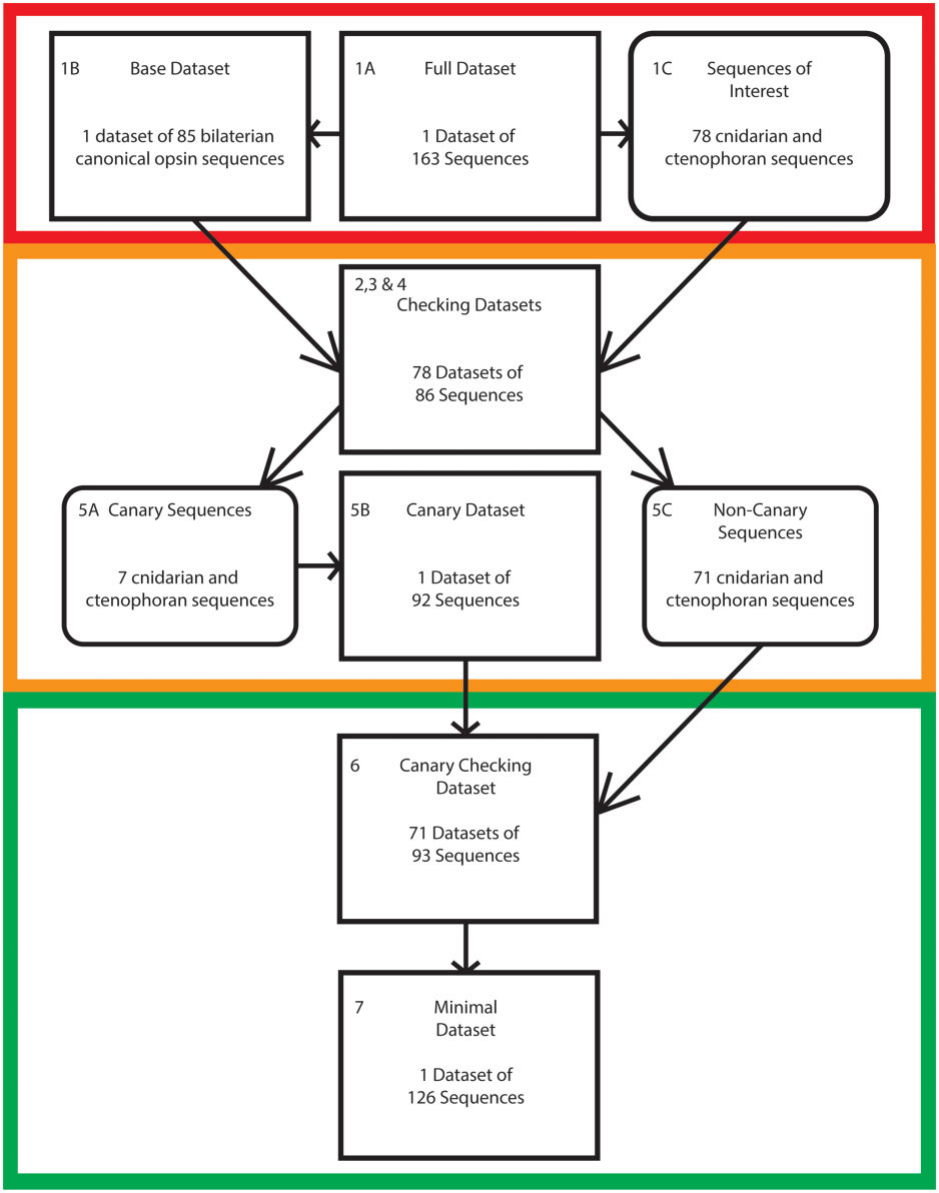
—The canary sequence methodology applied to the opsin data set. The number of sequences at each stage of the canary sequence approach, when applied to the nonbilaterian opsin sequences. Each stage is color-coded to correspond to the stages depicted in figure 2.

We assembled a data set of 98 well-characterized bilaterian opsins—downloaded from the NCBI website. This data set was assembled to avoid biasing the taxonomic composition of our data set in favor of groups that are overrepresented in sequence databases, such as the Vertebrata in the ciliary opsins, and the Arthropoda in the rhabdomeric opsins (see Heath et al. 2008 for more details). Our data set included sequences sampled from all bilaterian C, R, and Group 4. We did not include bilaterian sequences from recently proposed opsin families: Xenopsins, chaopsins and bathyopsins (Ramirez et al. 2016) as these families invariably share common ancestors with another canonical bilaterian opsin family (Ramirez et al. 2016), and therefore the order of the most basal duplications in the opsin family is fully defined by the order in which the C, R, and Group 4 opsins emerge. To this core group of sequences, we added opsins from nonbilaterian lineages sampled from three recent studies: Feuda et al., (2012, 2014), Ramirez et al., (2016), Schnitzler et al., (2012), for a total of 115 sequences—note that these sequences might include nonbilaterian representatives of the noncanonical opsin families. When sequences that were identical between the data sets were removed, the number of sequences retained dropped to 78; of these sequences, 5 belong to the Ctenophora, and 73 to the Cnidaria. Opsin sequences from Hering and Mayer (2014) were not directly considered, as all the sequences in that study were included in at least one of the other three considered data sets. The 78 ctenophoran and cnidarian sequences constitute our “sequences of interest” (see figs. 2 and 3), whereas the 20 bilaterian opsin sequences considered constitute our “base data set.” The “full data set” comprised all 98 considered sequences: The “base data set” plus the “sequences of interest” (as in point 1 of the “The Canary Sequence Approach to Identify Problematic Sequences” section). Opsin sequence alignments were generated using MUSCLE (Edgar 2004) and phylogenetic analyses were performed under the GTR+G (see Feuda et al. 2012, 2014; Vöcking et al. 2017 for the rationale) model in Phylobayes 3 (Lartillot et al. 2009). Comparisons of the maximum discrepancies observed over the bipartitions and the effective sample size in bpcomp and tracecomp (which are included in the Phylobayes distribution) was used to assess convergence. For all analyses two independent chains were run, and a burnin of 50% of the sample size was used, sampling every fiftieth tree following the burnin period. All alignments and Newick tree files of the canary sequence methodology are available at: https://bitbucket.org/flemingj/canarysequencemethodology (last accessed February 2, 2020).

## Results and Discussion

### The Canary Approach Correctly Identifies Ecdysozoa Monophyly Using the Aguinaldo et al. (1997) Data Set

In supplementary figure S2, Supplementary Material online, we show that the canary sequence approach can be applied to Aguinaldo et al. (1997) data set to recover a monophyletic Ecdysozoa. The Aguinaldo et al. (1997) data set is composed of 18s rRNA sequences—some of the Nematode representatives in this data set are long branched and attracted to the root of the tree (Holton and Pisani 2010) under certain analytical conditions. This is a well understood problem that produces a known and replicable phylogenetic artefact when analyzed using poorly fitting substitution models. We followed the protocol in figure 2 and points 1–6 of the “The Canary Sequence Approach to Identify Problematic Sequences” section to identify “canary” and “noncanary sequences of interest” and to ultimately remove all “potentially problematic” sequences in this data set. Two sequences emerged as canary sequences: The 18s rRNA sequences for *Caenorhabditis* and *Trichuris*. One sequence emerged as “potentially problematic”: The *Strongyloides* 18S rRNA sequence. The “minimal tree” that excludes the *Strongyloides* 18S rRNA sequence recovered monophyletic Ecdysozoa (see supplementary fig. S2, Supplementary Material online).

### The Canary Method Correctly Resolves Platyhelminthes Using the Carranza et al. (1997) Data Set

To more firmly assess the capabilities of the canary approach, a second data set was analyzed—Carranza et al. (1997). Carranza et al. (1997) undertook a study of eighteen 18S rRNA “flatworm” sequences (3 Acoela and 15 Platyhelminthes). They found a monophyletic Platyhelminthes separated from a monophyletic Acoelomorpha. Acoelomorpha emerged as the sister to the other Bilateria (but not in all their analyses). However, they failed to recover a monophyletic Lophotrochozoa, inclusive of the catenulid flatworms. However, current molecular consensus indicates that Platyhelminthes are a monophyletic member of the Lophotrochozoa (Halanych 2004), with the position of the Acoelamorpha still being disputed (e.g., Philippe et al. 2019). We focused on the “flatworms” (Platyhelminthes plus Catenulida), which we considered to be our “sequences of interest.” We followed the protocol in figure 2 (and points 1–6 above) to identify “canary sequences” and “noncanary sequences of interest” from our flatworm sequences. Only the 18S rRNA of *Discocelis tigrina* was found to be a canary sequence, and of the noncanary sequences of interest, only the 18S rRNA of *Planocera* emerged as “not problematic.” A “minimal data set” (see fig. 2) was derived including these two flatworm sequences only (*Planocera* and *D.* *tigrina)*, and the minimal tree derived from this dataset recovered monophyletic Platyhelminthes, and Lophotrochozoa, in accordance with current molecular consensus (see supplementary fig. S3, Supplementary Material online).

### Simulation Data Sets

We applied the canary approach (figure 2 and points 1–6 of the “The Canary Sequence Approach to Identify Problematic Sequences” section) to 50 simulated data sets (see supplementary information, Supplementary Material online, for data sets). We found that the canary approach has a 66% success rate against our relevant data sets. While a 66% success rate is not overwhelmingly high, it should be noted that 1) we are aware of no other approaches that are available to identify problematic sequences in single-gene analyses, and that 2), in the 34% of the cases where the method did not improve the analytical result, failure of the canary approach was caused by its inability to identify and thus exclude problematic sequences. Accordingly, the canary approach seems conservative and, based on our current set of results, when it fails, it is not because it identifies false positives (i.e., it does not seem to erroneously identify non problematic sequences as “potentially problematic”). Accordingly, even in the worst case scenario, the application of the canary method does not seem to lead to results that are worse than those that would have been obtained if the method was not applied.

With reference to individual sequences, *Caenorhabditis elegans* 18S was not rejected in the original data set. However, the sequences simulated to represent this taxon were rejected in 57.5% of the simulations. This result reflects the fact that we exacerbated branch lenghts in our simulation settings.. Sequences simulated to represent *Strongyloides stercorali* (which was rejected in the original data set) were rejected in 63.6% of the simulations. 78.8% of the successful simulations reject at least one of these two simulated sequences, with the remaining sequences being able to resolve a correctly positioned monophyletic Nematoda. As the canary sequence approach scales with the capabilities of the models used to resolve the “checking tree” and “canary checking tree,” better results could be expected in simulation using more sophisticated models that were not used here to maintain comparability with the original results of Aguinaldo et al. (1997).

### Identifying Problematic Nonbilaterian Opsin

We sampled 115 cnidarian and ctenophoran sequences from Schnitzler et al. (2012) (19 sequences), Feuda et al. (2014) (31 sequences), and Ramirez et al. (2016) (65 sequences). Of these sequences, 37 were found to be identical (the same sequence but possessing different names between the data sets) leaving a total of 78 nonbilaterian opsins (sequences of interest) and 85 bilaterian opsins (base data set). The canary approach found 37 of the 78 nonbilaterian opsin sequences to be problematic (see supplementary table S1, Supplementary Material online, for further details). Of the 37 discarded, 10 were present in Feuda et al. (2014), 32 in Ramirez et al. (2016), and 10 in Schnitzler et al. (2012). The starlet sea anemone *Nematostella vectensis* provided the highest number of sequences of interest, but also the highest number of problematic sequences, whereas the anthomedusan *Cladonema radiatum* and the box jellyfish *Tripedalia cystophora* provided the largest proportion of nonproblematic sequences. Only two of eight opsins were problematic for *C.* *radiatum*, whereas 5 of 18 opsins were problematic in the case of the box jellyfish (see supplementary table S1, Supplementary Material online).

### The “Minimal” Opsin Tree

**Fig. 4.**
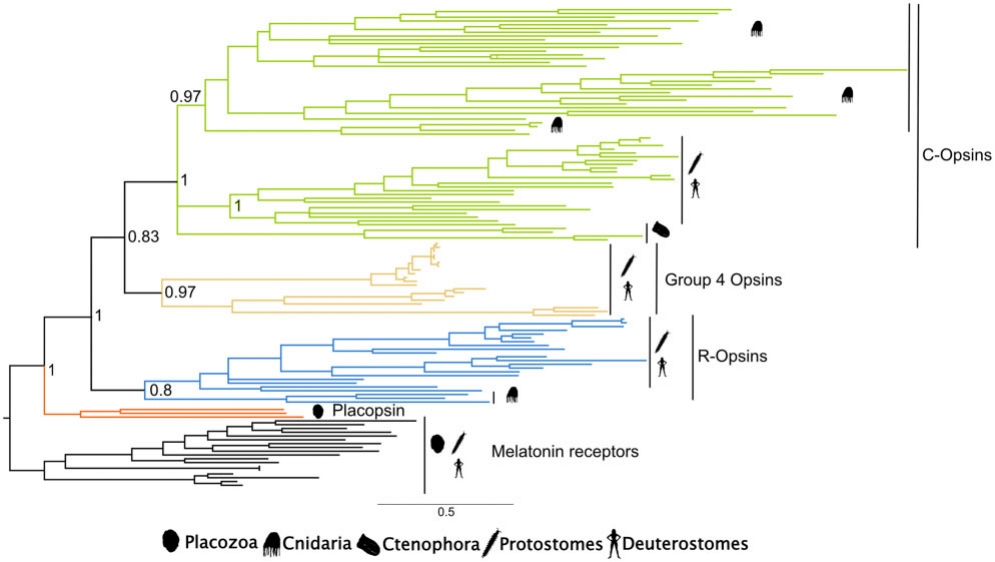
—The Minimal opsin tree recovered under GTR+G. Support values (Bayesian PPs) are reported only for key nodes.

**Fig. 5.**
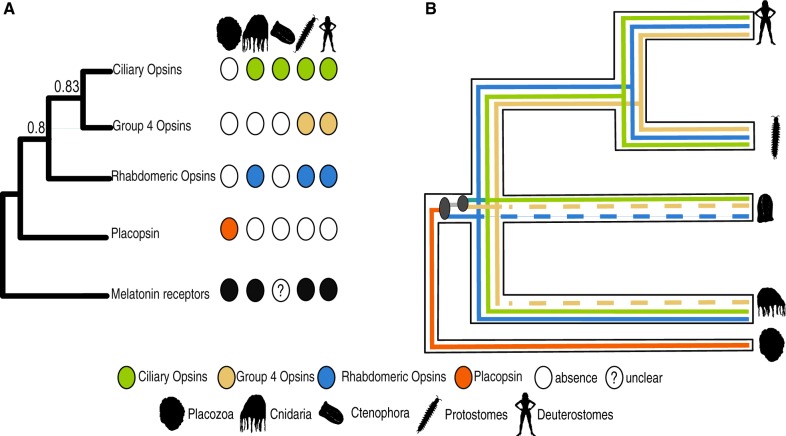
—Synopsis of opsin evolution. (*A*) Phylogenetic distribution of canonical opsin in Eumetazoans. (*B*) Duplication pattern of opsin genes in Eumetazoa. Dashed lines indicate lineage-specific losses.

Once “potentially problematic” cnidarian and ctenophoran sequences were excluded from the analyses, the “minimal opsin tree” showed that the remaining nonbilaterian opsins were related to two groups: The rhabdomeric opsins and the ciliary opsins (figs. 4 and 5). More precisely, nonbilaterian sequences that in Ramirez et al. (2016) emerged as xenopsins (sharing a common ancestor with the group 4 opsins—see fig. 1) and as “canonical cnidarian visual opsins” (sharing a common ancestor with the ciliary and rhabdomeric opsins—fig. 1) were all recovered as sharing a common ancestor with the bilaterian ciliary opsins. In Feuda et al. (2014) these sequences resolve as members of either the group 4 opsins or the ciliary opsins. In Schnitzler et al. (2012), these same sequences either emerge as group 4 opsins or as the sister of both the group 4 and ciliary opsins. Our “Minimal opsin tree” has elements in common with the trees of Feuda et al. (2014), Ramirez et al. (2016), and Schnitzler et al. (2012), whereas also differing from all of these trees, suggesting some sort of consensus solution instead. Cnidarian sequences that are resolved as rhabdomeric in our minimal opsin tree also emerged as rhabdomeric in Feuda et al. (2012, 2014), whereas in Schnitzler et al. (2012) these sequences emerged as the sister group of all the other opsins. In Ramirez et al. (2016) these same nonbilaterian opsins emerged as members of the newly proposed chaopsins group, which is suggested to share a common ancestor with the group 4 opsins, together with four echinoderm opsins.

Cnidarian and ctenophoran group 4 opsins are not recovered in our minimal opsin tree. Accordingly, our results suggest either an independent loss of the group 4 opsins in the nonbilaterians or that all nonbilaterian group 4 opsin sequences are problematic according to the canary approach. The latter hypothesis is supported by the fact that both Schnitzler et al. (2012) and Feuda et al. (2014) recovered cnidarian and ctenophoran sequences within the group 4 opsins that were identified as problematic by the canary sequence approach. However, the suggestion of a real loss of the group 4 opsins within nonbilaterians is supported by Ramirez et al. (2016), in which sequences recovered as group 4 opsins by the previously cited studies were instead recovered as members of noncanonical opsin families. In any case, it is clear that the presence of group 4 opsins in non-Bilateria deserves further investigation.

Two particularly important nonbilaterian opsins are mnemiopsis3 and acropsin3. The first was found at the root of the opsin tree in Schnitzler et al. (2012), in presumed agreement with the Ctenophora-sister hypothesis. However, Feuda et al. (2014) suggested that the placement was a phylogenetic artefact and that this sequence was more likely linked to the group 4 opsins. Here, we found mnemiopsis3 to be problematic, and thus likely to be involved in the generation of tree reconstruction artefacts. This conclusion is in accordance with Feuda et al. (2014). However, as this sequence was removed by the canary sequence method we could not confirm this sequence as a Group 4 Opsin.

Acropsin3 was found by Mason et al. (2012) to link to a G-protein of the Gq type (as expected from rhabdomeric opsins), and there is thus biochemical evidence suggesting that this protein might be a rhabdomeric opsin. Indeed, Feuda et al. (2014) found acropsin3 to be a rhabdomeric opsin nesting with two more sequences from *Nematostella* that Feuda et al. (2012) and Suga et al. (2008) previously resolved as cnidarian rhabdomeric opsins. However, Ramirez et al. (2016) found these sequences to be the sister of both the ciliary and rhabdomeric opsins, raising doubts about whether cnidarian rhabdomeric opsins exist.

Acropsin3 emerged as a canary sequence in our study. This suggests that its position might be affected by the inclusion of problematic sequences in the data set. The application of the canary approach suggested that the putative *Nematostella* rhabdomeric opsins of Feuda et al. (2012, 2014) are problematic and could have had a negative impact also on the placement of acropsin3 in Feuda et al. (2014). However, also in the minimal opsin tree, which excludes all potentially problematic sequences, acropsin3 emerged (together with two more non problematic *Nematostella* sequences) as a rhabdomeric opsin, strengthening the evidence for the existence of this opsin type in Cnidaria (Suga et al. 2008; Feuda et al. 2012, 2014) and further suggesting that cnidarians might possess rhabdomeric opsins (fig. 5).

## Conclusions

We develop a method that can identify potentially problematic sequences in single-gene data sets. We validated the test using case studies and simulation and then applied it to the problem of opsin evolution. While we investigated the removal of potentially problematic sequences from the data set, it is clear that such sequences could be retained, and we do not necessarily advocate their exclusion from an analysis. If one was to retain all the sequences from a data set, the result of the canary pipeline would still be useful, as knowledge of which sequences in the data set are “potentially problematic,” and which are “canary sequences” (i.e., unstable but not necessarily problematic) would still be useful when interpreting phylogenetic results. A practical example of this would be that of mnemiopsis3. Feuda et al. (2014) suggested that this sequence represents a ctenophoran group 4 opsin. If we were to retain mnemiopsis3 in our dataset, we could have confirmed that placement, but the canary approach would have still suggested that mnemiopsis3 does not provide conclusive evidence for the existence of group 4 opsins in Ctenophora. 

Our minimal opsin tree confirms that the three main canonical opsin lineages emerged before the separation of Cnidaria, Ctenophora, and Bilateria (fig. 5). Ctenophora possesses sequences that share a common ancestor with the bilaterian ciliary opsins, and the position of the ciliary opsins in the minimal opsin tree suggests that the shared ancestor of Ctenophora, Cnidaria, and Bilateria possessed three opsins. These opsins emerged from two duplications in the stem lineage subtending the crown defined by these taxa. Whether that lineage is the stem metazoan lineage or the stem eumetazoan lineage will depend on whether Porifera represent the sister group of all the other animals (Pisani et al. 2015; Feuda et al. 2017; King and Rokas 2017; Pett et al. 2019; Zhao et al. 2019) or not. Irrespective of that, according to our minimal opsin tree, the first duplication in opsin history separated the rhabdomeric opsins from the common ancestor of the ciliary and group 4 opsins. The second separated the ciliary opsins from the group 4 opsins (Feuda et al. 2012, 2014; Hering and Mayer 2014). Accordingly, we argue that the absence of rhabdomeric opsins in Ctenophora and of group 4 opsins in Cnidaria and Ctenophora can be attributed to either a secondary loss or a failure to unambiguously detect genes belonging to this opsin family. We suggest the latter possibility to be more likely.

## Supplementary Material

evaa015_Supplementary_Data

## Notes

**Data deposition:** This project has been deposited at https://bitbucket.org/flemingj/canarysequencemethodology.
