# Supplementary material for: A Novel Approach to Investigate the Effect of Tree Reconstruction Artifacts in Single-Gene Analysis Clarifies Opsin Evolution in Nonbilaterian Metazoans
Source: Genome Biol Evol. 2020 Feb 3;12(2):3906–16. doi: 10.1093/gbe/evaa015 (PMC7058159; doi:10.1093/gbe/evaa015)
Supplement: evaa015_Supplementary_Data [file evaa015_supplementary_data.zip › Supplmenentary_caption.pdf]

Figure S1. The Canary Sequence Approach in a full detailed flowchart. A more concise explanation of the process is available as Figure 2 of the manuscript, and the Approach is described in detail in the main body of the manuscript.

Figure S2. The minimal tree recovered by the application of the canary sequence approach to the Aguinaldo et al (1997) dataset. This tree was recovered in PhyML under the JC69 model.

Figure S3. The minimal tree recovered by the application of the canary sequence approach to the Carranza et al (1997) dataset. This tree was recovered in PhyML under the JC69 model nodes support is estimated using aLRTs.

Figure S4. The minimal opsin tree in full – the minimal opsin tree is additionally presented in a curated format as figure 4 of the manuscript. This tree was recovered in Phylobayes under the GTR+G model, nodes support is estimated using bayesian posterior probabiliyu.

Table S1. Cnidarian and ctenophoran sequences considered as part of this study. Compositional heterogeneity measures are given for sequences identified as potential canary sequences by virtue of their isomorphic topology.
