# Supplementary material for: A Novel Approach to Investigate the Effect of Tree Reconstruction Artifacts in Single-Gene Analysis Clarifies Opsin Evolution in Nonbilaterian Metazoans
Source: Genome Biol Evol. 2020 Feb 3;12(2):3906–16. doi: 10.1093/gbe/evaa015 (PMC7058159; doi:10.1093/gbe/evaa015)
Supplement: evaa015_Supplementary_Data [file evaa015_supplementary_data.zip › TableS1 (1).docx]

| Phylum | Species | Sequence Name | Canary Seq? | Problematic  Sequence? | Comp Hetero |
| --- | --- | --- | --- | --- | --- |
| Ctenophora |  |  |  |  |  |
|  | *Mnemiopsis leidyi* | Mnemiopsis_leidy1/1- |  | N |  |
|  |  | Mnemiopsis_opsin2_12 |  | N |  |
|  |  | Mnemiopsis_opsin3_21 |  | Y |  |
|  | *Pleurobrachia pileus* | Pleurobrachia_opsin1 | Y | N | 0.750 |
|  |  | Pleurobrachia_opsin2 |  | N |  |
|  |  |  |  |  |  |
|  |  |  |  |  |  |
| Cnidaria |  |  |  |  |  |
|  | *Nematostella vectensis* | NV_CN151Suga08_/1-24 |  | N |  |
|  |  | NV_CN146Suga08_/1-24 |  | N |  |
|  |  | NV_CN158Suga08_/1-24 |  | N |  |
|  |  | NV_CN152Suga08_/1-24 |  | Y |  |
|  |  | NV_CN144Suga08_/1-23 |  | Y |  |
|  |  | NV_CN145Suga08_/1-23 |  | Y |  |
|  |  | NV_CN137Suga08_/1-24 |  | Y |  |
|  |  | NV_CN143Suga08_/1-24 |  | Y |  |
|  |  | NV_CN135_Suga08/1-24 |  | Y |  |
|  |  | NEM_426957_jgi/1-22 |  | Y |  |
|  |  | NEM_445570_jgi/1-22 |  | Y |  |
|  |  | NEM_444070_jgi/1-22 |  | Y |  |
|  |  | Plos1_1/19-255 |  | N |  |
|  |  | Plos1_2/1-247 |  | N |  |
|  |  | Plos1_3/1-234 |  | N |  |
|  |  | A9UMW6_Nematostella_vectensis_opsin |  | N |  |
|  |  | Nematostella_95791 |  | Y |  |
|  |  | A9UMX0_Nematostella_vectensis_opsin |  | Y |  |
|  |  | Nematostella_131013 |  | Y |  |
|  |  | Nematostella_96290 |  | Y |  |
|  |  | Nematostella_214772 |  | Y |  |
|  |  | Nematostella_214772 |  | Y |  |
|  |  | Nematostella_199627 |  | Y |  |
|  |  | A9UMZ2_Nematostella_vectensis_opsin |  | Y |  |
|  |  | A9UMZ5_Nematostella_vectensis_opsin |  | Y |  |
|  |  | Nematostella_214775 |  | Y |  |
|  | *Cladonema radiatum* | ClR_CN168Suga08/1-19 | Y | N | 0.452 |
|  |  | ClR_CN116Suga08/1-23 | Y | N | 0.726 |
|  |  | ClR_CN120Suga08/1-23 | Y | N | 0.560 |
|  |  | ClR_CN101Suga08/1-24 |  | N |  |
|  |  | CR_CN100_Koyana/1-24 |  | N |  |
|  |  | A9CR26_Cladonema_radiatum_opsin |  | Y |  |
|  |  | A9CR28_Cladonema_radiatum_opsin |  | Y |  |
|  |  | A9CR38_Cladonema_radiatum_opsin |  | N |  |
|  |  | A9CR39_Cladonema_radiatum_opsin |  | N |  |
|  |  | A9CR43_Cladonema_radiatum_opsin |  | N |  |
|  |  | A9CR48_Cladonema_radiatum_opsin |  | N |  |
|  | *Hydra vulgaris* | HM_CN131_221128/1-20 | Y | N | 0.309 |
|  |  | HM_CN170_UPI000/1-23 |  | N |  |
|  |  | F1LIN8_Hydra_vulgaris_opsin |  | Y |  |
|  |  | F1LIN9_Hydra_vulgaris_opsin |  | N |  |
|  |  | F1LIP0_Hydra_vulgaris_opsin |  | N |  |
|  |  | F1LIP1_Hydra_vulgaris_opsin |  | Y |  |
|  |  | F1LIP2_Hydra_vulgaris_opsin |  | N |  |
|  |  | F1LIP3_Hydra_vulgaris_opsin |  | Y |  |
|  |  | F1LIP4_Hydra_vulgaris_opsin |  | Y |  |
|  |  | F1LIP5_Hydra_vulgaris_opsin |  | Y |  |
|  | *Acropora palmata* | acropsin2/1-248_no_c | Y | N | 0.095 |
|  |  | acropsin1/1-240_no_c |  | N |  |
|  |  | acropsin3/1-252_no_c | Y | N | 0.881 |
|  | *Acropora Digitifera* | 1_Acroporadigitifera_Acropora_digitifera_58percent_match_PREDICTED_visual_pigmentlike_receptor_peropsin |  | Y |  |
|  |  | XP_015763203_Acropora_digitifera_na |  | Y |  |
|  |  | XP_015773304_Acropora_digitifera_PREDICTED:_melanopsinlike |  | Y |  |
|  |  | XP_015776718_Acropora_digitifera_PREDICTED:_opsin3like |  | Y |  |
|  | *Podocoryna carnea* | A9CR57_Podocoryna_carnea_opsin |  | Y |  |
|  |  | A9CR60_Podocoryna_carnea_opsin |  | Y |  |
|  | *Tripedalia cystophora* | A0A059NTC5_Tripedalia_cystophora_Clike_opsin |  | N |  |
|  |  | A0A059NTC6_Tripedalia_cystophora_Clike_opsin |  | N |  |
|  |  | A0A059NTC7_Tripedalia_cystophora_Clike_opsin |  | N |  |
|  |  | A0A059NTC8_Tripedalia_cystophora_Clike_opsin |  | N |  |
|  |  | A0A059NTD1_Tripedalia_cystophora_Clike_opsin |  | Y |  |
|  |  | A0A059NTD2_Tripedalia_cystophora_Clike_opsin |  | N |  |
|  |  | A0A059NTD3_Tripedalia_cystophora_Clike_opsin |  | N |  |
|  |  |  |  |  |  |
|  |  | A0A059NTD4_Tripedalia_cystophora_Clike_opsin |  | N |  |
|  |  | A0A059NTD5_Tripedalia_cystophora_Clike_opsin |  | N |  |
|  |  | A0A059NTD6_Tripedalia_cystophora_Clike_opsin |  | Y |  |
|  |  | A0A059NTD7_Tripedalia_cystophora_Clike_opsin |  | Y |  |
|  |  | A0A059NTG1_Tripedalia_cystophora_Clike_opsin |  | N |  |
|  |  | A0A059NTG2_Tripedalia_cystophora_Clike_opsin |  | Y |  |
|  |  | A0A059NTG3_Tripedalia_cystophora_Clike_opsin |  | N |  |
|  |  | A0A059NTG7_Tripedalia_cystophora_Clike_opsin |  | Y |  |
|  |  | A0A059NTG8_Tripedalia_cystophora_Clike_opsin |  | N |  |
|  |  | A0A059NTG9_Tripedalia_cystophora_Clike_opsin |  | N |  |
|  |  | EU310498_Tripedalia_cystophora_copsinlike |  | N |  |
|  |  |  |  |  |  |
