# Supplementary figures and images for: A Novel Approach to Investigate the Effect of Tree Reconstruction Artifacts in Single-Gene Analysis Clarifies Opsin Evolution in Nonbilaterian Metazoans
Source: Genome Biol Evol. 2020 Feb 3;12(2):3906–16. doi: 10.1093/gbe/evaa015 (PMC7058159; doi:10.1093/gbe/evaa015)

# Figure S1

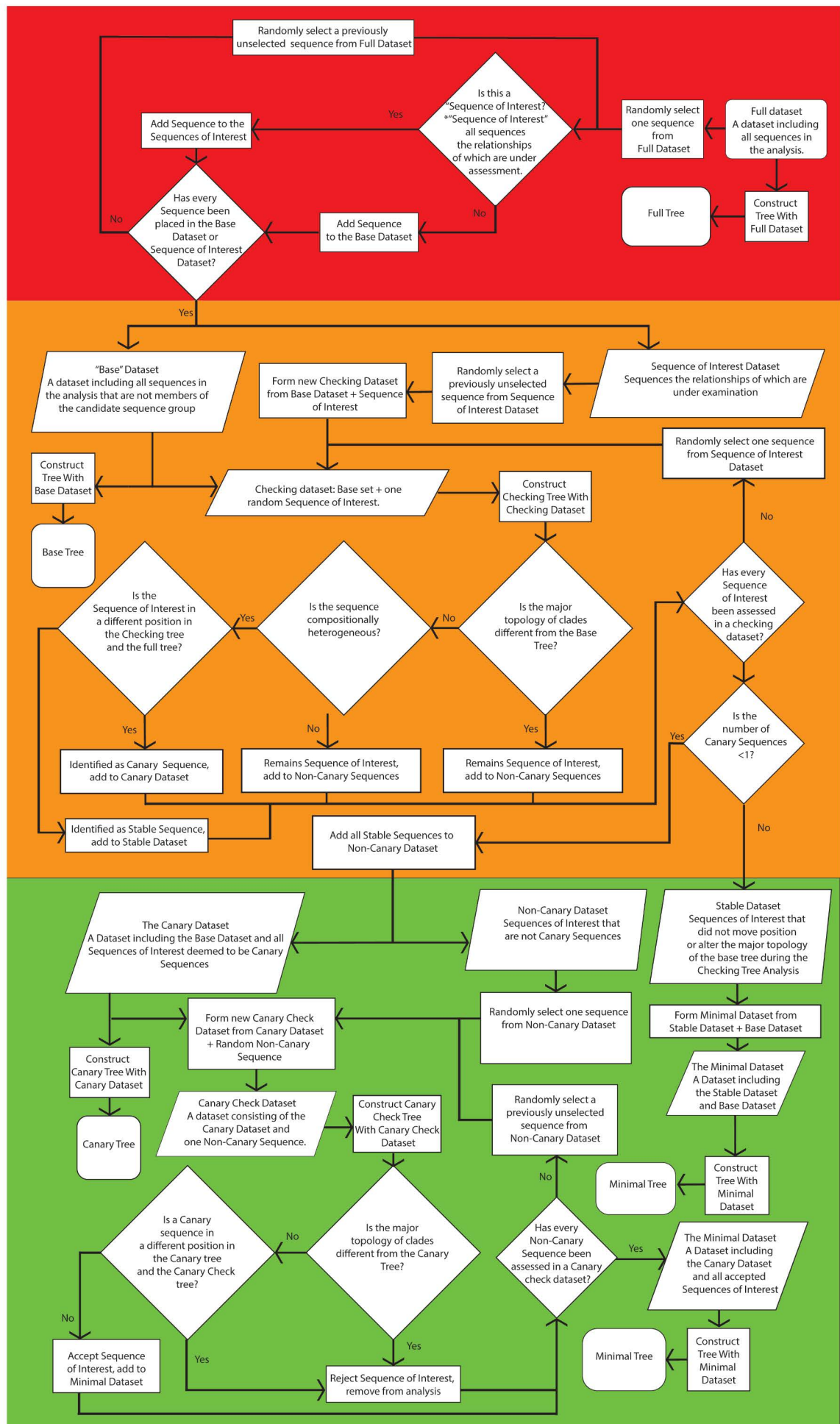

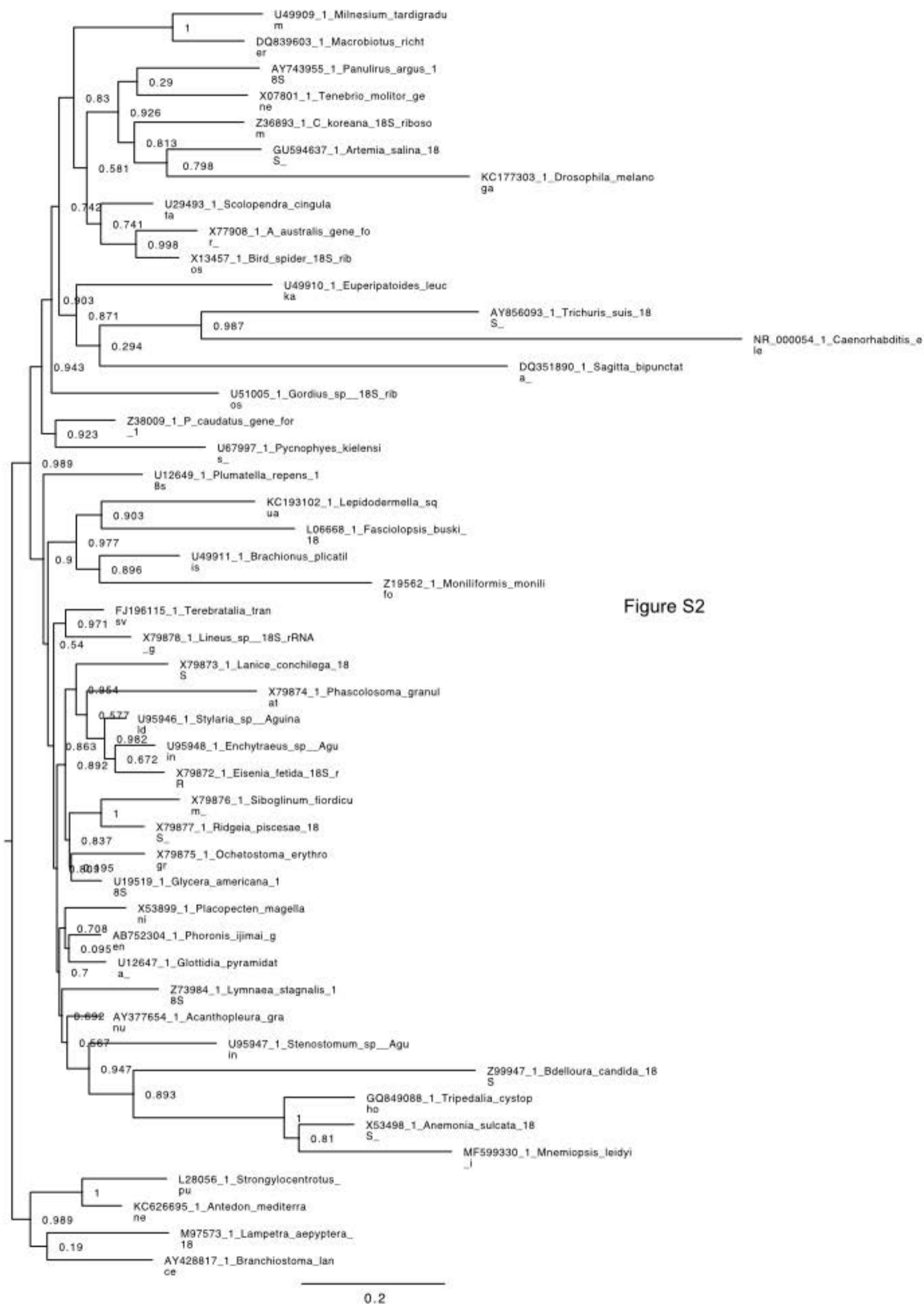

Figure S2

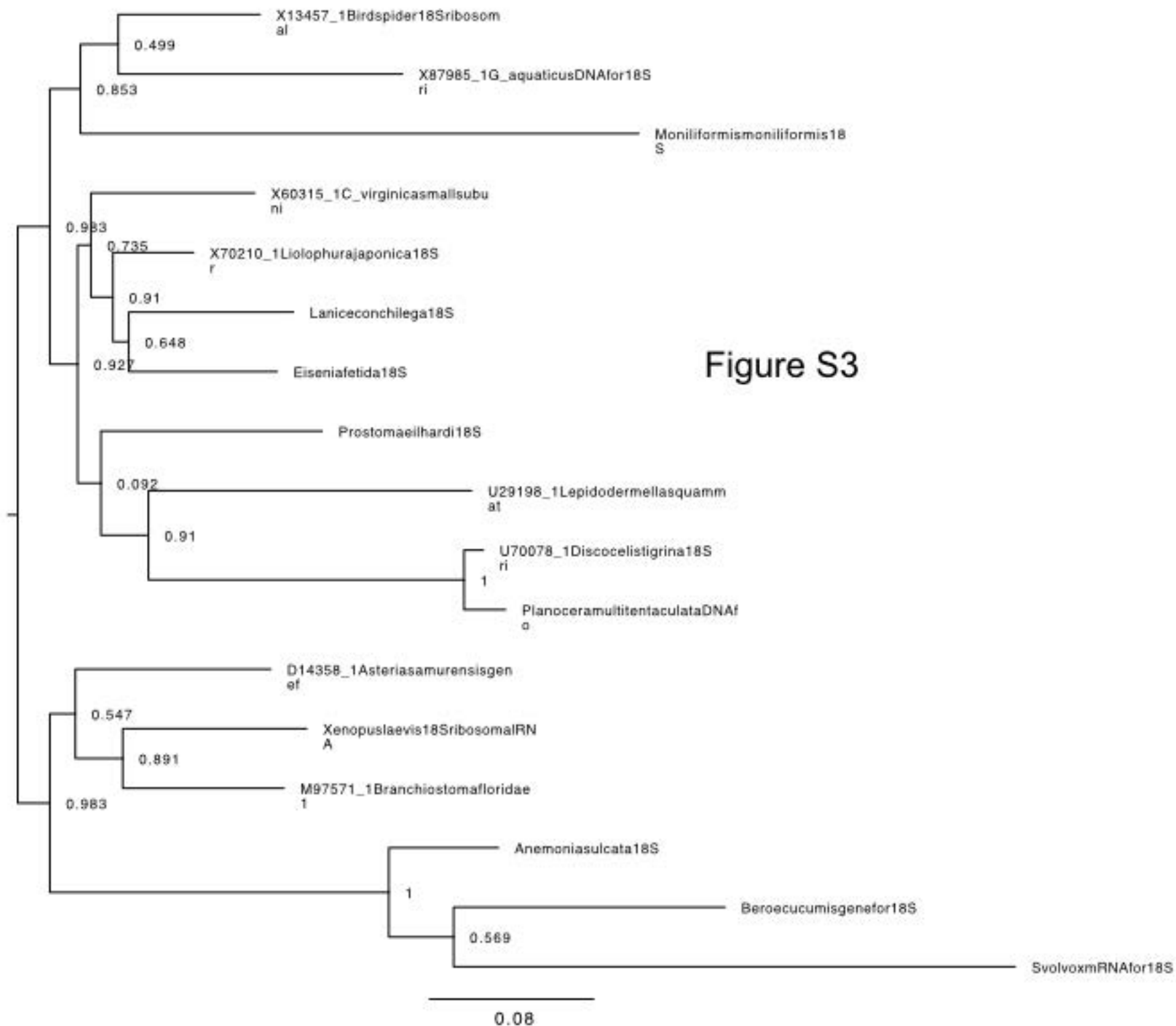

Figure S3

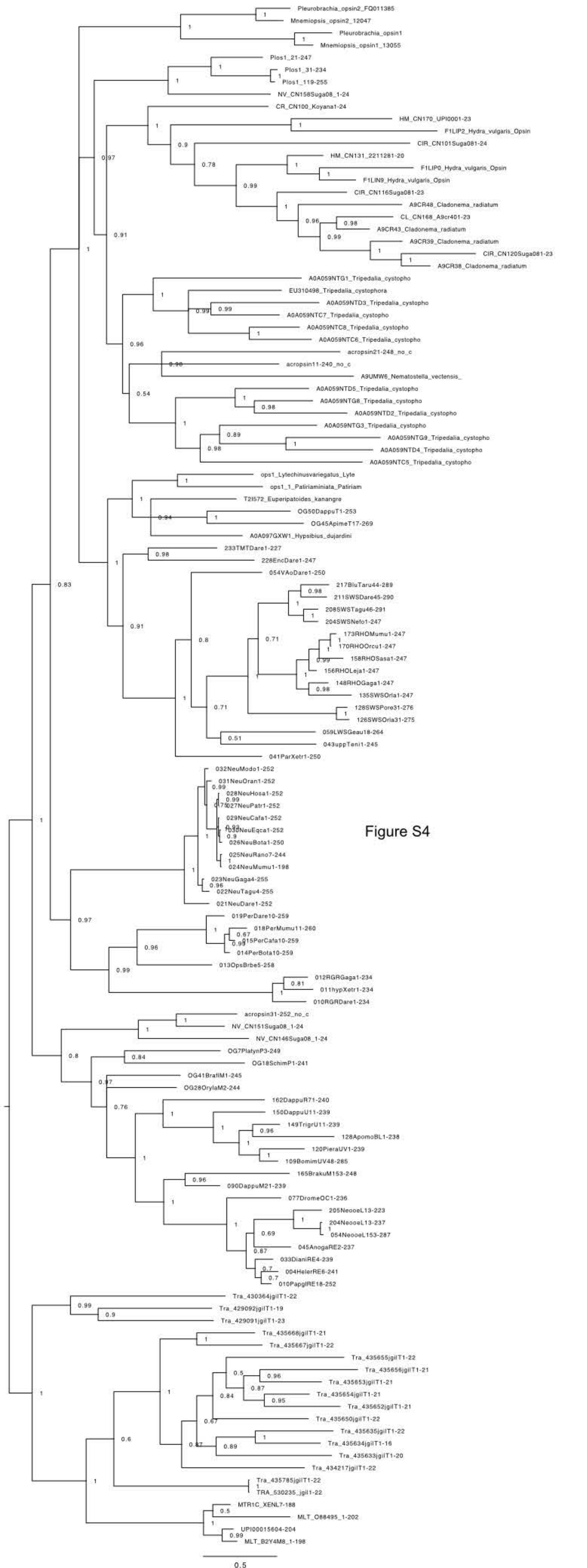

Figure S4

Supplement: evaa015_Supplementary_Data [file evaa015_supplementary_data.zip › Supplementary_Figures.pdf]
